# Lipid and hyperglycemia factors in first‐ever penetrating artery infarction, a comparison between different subtypes

**DOI:** 10.1002/brb3.694

**Published:** 2017-05-10

**Authors:** Shaoyang Sun, Yanqiang Wang, Yuge Wang, Xuejiao Men, Jian Bao, Xueqiang Hu, Zhengqi Lu

**Affiliations:** ^1^ Department of NeurologyThe Affiliated Hospital of Qingdao UniversityQingdaoChina; ^2^ Department of NeurologyThe Affiliated Hospital of Wei fang Medical UniversityWeifangChina; ^3^ Department of NeurologyThe Third Affiliated Hospital of Sun Yat‐sen UniversityGuangzhouChina

**Keywords:** early neurological deterioration, Intracranial branch atheromatous disease, lipohyalinotic degeneration, low‐density lipoprotein cholesterol, penetrating artery infarction

## Abstract

**Background:**

The pathogenesis and progression of branch atheromatous disease (BAD), which differs from lipohyalinotic degeneration (LD), remains controversial. Few studies have investigated the lipid indices and glycometabolism status factors for BAD in first‐ever penetrating artery infarction (PAI).

**Methods:**

We retrospectively examined acute stroke patients with PAI admitted within 3 days after stroke. All patients underwent diffusion weight magnetic resonance imaging (DWI) and magnetic resonance angiography (MRA) and/or computed tomography angiography (CTA). Progression was defined as an increase by 2 point or higher in the National Institutes of Health Stroke Scale score. The characteristics, clinical data were statistically analyzed.

**Results:**

BAD and LD were diagnosed in 142 (57%) and 107 (43%) patients, respectively. Patients with BAD had higher low‐density lipoprotein cholesterol (LDL‐C) compared with those with LD (*p *= .013). Elevated LDL‐C was related to early neurological deterioration in patients with BAD (*p *= .045). The percentage of lenticulostriate arterial (LSA) infarction was greater than that of the pontine penetrating arterial (PPA) infarction in acute PAI (75.1% vs. 24.9%; *p* < .001). PPA infarction was more prevalent in the BAD group compared with the LD group (34.5% vs. 12.1%, *p *< .001). The PPA infarction had older age at onset and higher HbA1c concentrations than those with the LSA infarction (*p *= .014, *p *= .036 respectively) in the BAD and LD patients, respectively.

**Conclusion:**

LDL‐C may be associated with both the pathogenesis and progression of intracranial BAD. The LSA infarction was the most frequently subtypes in PAI. Age at onset and HbA1c seem to be closely associated with the PPA infarction of first‐ever PAI.

## 1 Introduction

Small deep brain infarct because of occlusion of one single penetrating artery was termed lacunar infarct in 1965 (Fisher, 1965). This classical concept of lacunar infarct persisted for decades. In 1989, according to different pathological changes, Caplan improved the theory and proposed a new type of ischemic cerebrovascular disease leading to an isolated deep brain infarction: branch atheromatous disease (BAD), which referred to the occlusion or stenosis of the proximal end of one penetrating artery based on atherosclerosis (Caplan, 1989). The concept of BAD, differing from lacunar infarction, which referred to the lipohyalinotic degeneration (LD) of the distal end of a penetrating artery, has recently been accepted in scientific research and clinical practice. In the Chinese ischemic stroke subclassification launched in 2011, both BAD and LD are classified as penetrating artery disease (PAD) (Gao, Wang, Xu, Li, & Wang, 2011). To develop more effective therapeutic strategy and secondary prevention of penetrating artery infarction (PAI), a better understanding of its risk factors and pathogenetic mechanisms will be helpful.

Several recent studies have reported, BAD tends to occur with more severe neurological deficits, and is more likely to undergo progression than LD (Baumgartner, Sidler, Mosso, & Georgiadis, 2003; Yamamoto et al., 2010, 2011). The risk factors, including male gender, diabetes mellitus, and intracranial atherosclerosis were related to pontine penetrating arterial (PPA) infarction in the BAD. Our research has reported the inflammatory factors, homocysteine (Hcy), and C‐reactive protein (CRP) were associated with progression and prognosis of BAD. Accumulating data suggest that lipids are central to the development of atherosclerotic plaques, and the oxidation of low‐density lipoprotein (LDL) is thought to play a critical role in the initiation of atherosclerosis (Matsuura, Lopez, Shoenfeld, & Ames, 2012; Rost et al., 2001; Tabuchi et al., 2007; Vila, Castillo, Davalos, & Chamorro, 2000; Yilmaz, Arumugam, Stokes, & Granger, 2006), low‐density lipoprotein cholesterol levels were associated with increased intracranial atherosclerotic stenosis (ICAS) (Park, Hong, Lee, Lee, & Kim, 2011). However, little attention has been paid to the impact of lipid indices and glycometabolism status in small artery disease especially in acute penetrating artery infarction.

## 2 Materials and Methods

### 2.1 Patients

**Figure 1:**
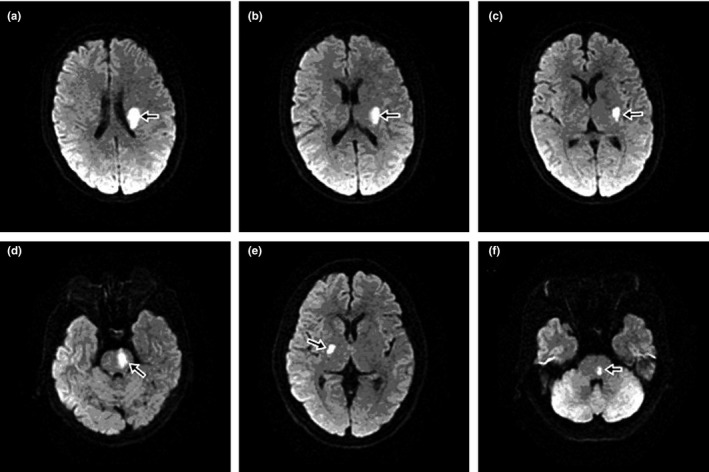
DWI findings in axial section (infarcts indicated by black arrow). (a, b, c) BAD in LSA territory. (d) BAD in PPA territory. (e) LD in LSA territory. (f) LD in PPA territory

A succession of 1458 inpatients with acute ischemic stroke (≥18 years old, <3 days of onset) whose focal neurologic deficits lasted over 24 hr was included in this study. All patients were admitted to the Department of Neurology, Third Affiliated Hospital of Sun Yat‐sen University from January 2008 to February 2012. Patients with PAD were screened out based on the lesions observed using head diffusion‐weighted magnetic resonance imaging (DWI; with one slice thickness of 5 mm), magnetic resonance angiography (MRA) and/or computed tomography angiography (CTA). The included cases had to meet the criteria of an isolated infarct in a clinically relevant territory of one penetrating artery, regardless of the size of infarct (Gao et al., 2011). BAD in the lenticulostriate artery (LSA) territory was defined as a supratentorial lesion >15 mm in diameter or visible for three or more axial slices; BAD in the paramedian pontine artery (PPA) territory was defined as a unilateral lesion extending to the ventral surface of the pons. LD was defined as an infarction with diameter <15 mm in the LSA territory, or an isolated infarction confined to the pontine parenchyma in the PPA territory (Yamamoto et al., 2011) (Figure 1).

### 2.2 Exclusion criteria

**Figure 2:**
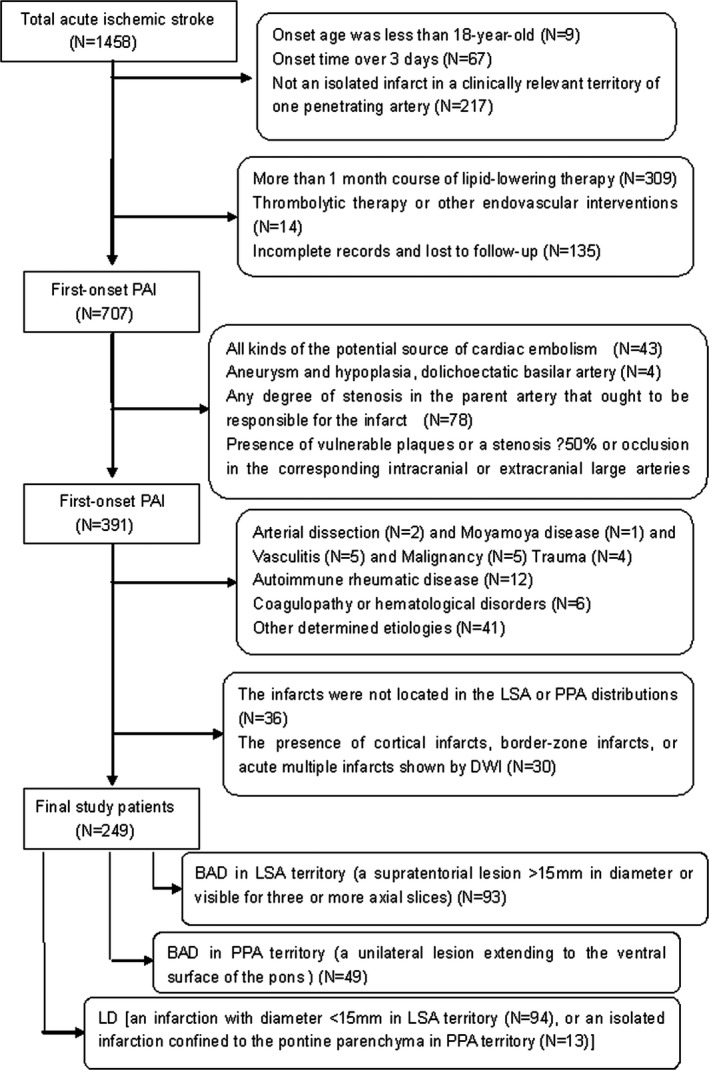
Study patients selection

The exclusion criteria included: (1) prior history of stroke or TIA and other causes of cerebral infarction, a potential source of cardiac embolism; (2) any degree of stenosis in the parent artery that ought to be responsible for the infarct; (3) the presence of vulnerable plaques or a stenosis ≥50% or occlusion in the corresponding intracranial or extracranial large arteries, such as middle cerebral artery and internal carotid artery; (4) the presence of cortical infarcts, border zone infarcts, or acute multiple infarcts shown by DWI; (5) the infarcts were not located in the LSA or PPA distributions; (6) a history of thrombolytic therapy or other endovascular interventions; (7) a history of arterial dissection, Moyamoya disease, vasculitis, autoimmune rheumatic disease, malignancy, trauma, coagulopathy, or hematological disorders; (8) a history of long‐term (≥1 month) statin therapy before admission; (9) some basilar artery diseases such as dissection, aneurysm, and hypoplasia, dolichoectatic basilar artery, embolism, vasospasms, and (10) incomplete records and follow‐up (Figure 2).

### 2.3 Clinical data

In all the selected patients, these were treated with anti‐platelet aggregation, improved recurrent, and other routine treatment. They have not received thrombolytic therapy or other endovascular interventions. A computed tomography scan was performed within 24 hr, and magnetic resonance imaging was performed within 72 hr after admission. MRI investigations included diffusion‐weighted imaging (TR: 6000 ms/TE: 61.5 ms), T2‐flair (TR: 8802 ms/TE: 129 ms), T2‐weighted (TR: 4800 ms/TE: 100 ms) and magnetic resonance angiography (TR: 27 ms/TE: 6.9 ms), which were obtained using a GE 1.5T MR scanner (General Electric, Milwaukee, WI, USA). Fasting (no caloric intake for at least 8 hr) venous blood samples were obtained within 24 hr after admission. Each patient accepted carotid ultrasonography, magnetic resonance angiography (MRA), or computed tomography angiography (CTA), 24 hr Holter monitoring, and other routine examinations during hospitalization. All brain images were analyzed by two neuroradiologists blinded to clinical information independently. The National Institutes of Health Stroke Scale (NIHSS) was performed daily to trace the disease course within the first 5 days. Progression was defined as a > 2‐point increase in the National Institutes of Health Stroke Scale for motor function during observation (Kwan & Hand, 2006). Basic clinical data, including age, gender, and NIHSS scores on admission and within 5 days, were collected from the patients’ records (Lind, Vessby, & Sundstrom, 2006).

### 2.4 Ethics statement

This study was approved by the local Ethics Committee of the Third Affiliated Hospital of Sun Yat‐sen University. Informed consent for this study was obtained from all patients or their family members.

### 2.5 Statistical analysis

Data from the selected patients were compared between the different groups. We used the *t*‐test for analyzing normally distributed variables and the Mann–Whitney *U* test for non‐normally distributed variables. The Pearson's *Χ* ^2^ test was used for categorical variables. The risk factors of ischemic stroke patients with BAD and LD, were analyzed by univariate logistic regression analysis. A *p*‐value <.05 was considered statistically significant. All statistical analyses were performed using SPSS version 16.0 software (SPSS Inc, Chicago, IL, USA).

## 3 Results

### 3.1 Demographic and clinical characteristics of patients with PAD

As shown in Table 1, of 1458 consecutive patients with acute ischemic stroke, 249 cases were included in our study, 210 patients underwent MRI, 39 patients underwent CT and CTA. 84.34% patients had MRI, among whom 142 (57.0%) were diagnosed as BAD and 107 (43.0%) as LD. Compared with the LD group, patients with BAD had significantly higher levels of LDL‐C (*p *= .013) and LDL‐C/HDL‐C ratio (*p *= .036). Meanwhile, characteristics including age, gender, HbAlc, and other serum lipid indices did not differ between the two groups (Table 1).

**Table 1:** Baseline characteristics of patients with BAD and LD

	BAD (*N *= 142)	LD (*N *= 107)	*p*
Male (%)	95 (66.9)	72 (67.3)	.949
Age, years	62.47 ± 11.85	65.10 ± 11.05	.228
HbAlc, %	6.69 ± 1.88	6.42 ± 1.22	.387
TC, mmol/L	5.37 ± 1.42	5.24 ± 1.15	.598
TRIG, mmol/L	2.00 ± 0.91	2.10 ± 1.39	.654
HDL‐C, mmol/L	1.18 ± 0.27	1.17 ± 0.30	.947
LDL‐C, mm/L	3.81 ± 1.19	3.33 ± 0.98	.013^a^
LDL‐C/HDL‐C ratio	3.33 ± 0.97	2.96 ± 0.90	.036^a^
ApoAI, g/L	1.33 ± 0.28	1.29 ± 0.20	.420
ApoB, g/L	0.94 ± 0.28	0.95 ± 0.30	.961
ApoB/ApoAI ratio	0.73 ± 0.21	0.74 ± 0.22	.745

BAD, branch atheromatous disease; LD, lipohyalinotic degeneration; HbAlc, Hemoglobin Alc; TC, total cholesterol; TRIG, triglyceride; HDL‐C, high‐density lipoprotein cholesterol; LDL‐C, low‐density lipoprotein cholesterol; ApoAI, apolipoprotein AI; ApoB, apolipoprotein B.

a *p* < .05.

### 3.2 Association between LDL‐C and progression of BAD and LD

Patients with BAD showed a significantly higher progressive ratio (39.4%) compared with those with LD (9.3%) (*p *< .001). The concentration of LDL‐C was significantly higher in patients with progressive BAD (4.23 ± 1.39 mmol/L) compared with those with non‐progressive BAD (3.66 ± 1.05 mmol/L) (*p *= .045); whereas, gender, age, HbA1c, and other lipid indices were not significantly different between the the progressive and non‐progressive patients in the BAD group (Table 2). Table 3 shows the logistic regression analysis results. In the univariate logistic analysis, LDL‐C level (more than 4.14 mmol/L) (OR = 1.96, *p *= .01) were found to be more strongly correlated with the progressive than non‐progressive patients in the BAD group. NIHSS score at admission (more than 3 points) (OR = 1.865, *p *= .036) were found to be more strongly correlated with the progressive than non‐ progressive patients in the LD group (Table 3).

**Table 2:** Characteristics of progressive and non‐progressive patients with BAD and LD

	BAD		*p*	LD		*p*
	Progressive	Non‐progressive		Progressive	Non‐progressive	
Number (%)	56 (39.4)^b^	86 (60.6)		10 (9.3)	97 (90.7)	
Male (%)	34 (60.7)	61 (70.9)	.206	8 (80.0)	64 (66.0)	.585
Age, years	63.37 ± 12.03	61.11 ± 11.69	.326	69.14 ± 14.94	64.45 ± 10.53	.302
HbAlc, %	7.57 ± 2.89	6.37 ± 1.24	.140	6.73 ± 1.24	6.37 ± 1.23	.470
TC, mmol/L	5.70 ± 2.07	5.26 ± 1.11	.416	5.50 ± 0.84	5.21 ± 1.19	.532
TRIG, mmol/L	1.90 ± 0.85	2.21 ± 1.04	.157	2.20 ± 1.43	2.07 ± 1.52	.708
HDL‐C, mmol/L	1.16 ± 0.38	1.21 ± 0.23	.545	1.26 ± 0.33	1.16 ± 0.29	.285
LDL‐C, mmol/L	4.23 ± 1.39	3.66 ± 1.05	.045^a^	3.62 ± 0.70	3.29 ± 0.92	.361
LDL‐C/HDL‐C ratio	3.64 ± 1.04	3.20 ± 0.93	.142	3.05 ± 1.02	2.95 ± 0.87	.774
ApoAI, g/L	1.40 ± 0.41	1.31 ± 0.22	.260	1.38 ± 0.23	1.27 ± 0.19	.101
ApoB, g/L	0.99 ± 0.41	0.94 ± 0.23	.595	0.97 ± 0.35	0.95 ± 0.28	.849
ApoB/ApoAI ratio	0.74 ± 0.31	0.73 ± 0.17	.826	0.69 ± 0.22	0.75 ± 0.22	.482

BAD, branch atheromatous disease; LD, lipohyalinotic degeneration; HbAlc, Hemoglobin Alc; TC, total cholesterol; TRIG, triglyceride; HDL‐C, high‐density lipoprotein cholesterol; LDL‐C, low‐density lipoprotein cholesterol; ApoAI, apolipoprotein AI; ApoB, apolipoprotein B.

a *p* < .05.

b *p* < .001 compared with progressive patients with LD.

**Table 3:** Multivariate logistic regression analysis of progressive patients with BAD and LD

	Progressive with BAD			Non‐progressive with LD		
	Odds ratio (OR) value	95% CI	*p*	Odds ratio (OR) value	95% CI	*p*
LDL‐C (≥4.14 mmol/L)	1.960	1.162–2.901	.01^a^	1.361	0.815–2.277	NS
NIHSS score at admission (≥3 min)	1.109	0.673–1.797	NS	1.865	1.015–3.230	.036^a^

BAD, branch atheromatous disease; LD, lipohyalinotic degeneration; OR, Odds ratio; CI, Confidence Interva; LDL‐C, low‐density lipoprotein cholesterol; NIHSS, National Institutes of Health Stroke Scale; NS, no statistical difference.

a *p* < .05.

### 3.3 Association between onset age and HbA1c and subgroups of BAD

Table 2 shows the characteristics of subgroups with BAD. We divided all the cases into the LSA (*n *= 187) and PPA groups (*n *= 62) based on the different locations of infarcts. The percentage of LSA infarction was greater than that of the PPA infarction in acute PAI (75.1% vs. 24.9%; *p* < .001). Pontine infarctions were more prevalent in the BAD group compared with the LD group (34.5% vs. 12.1%, *p* < .001). In the BAD group, the patients with PPA infarctions had older age at onset than those with the LSA infarctions (*p* = .014). Moreover, among patients with LD, a significantly higher HbA1c concentration was observed in the PPA group (*p* = .036). (Table 4).

**Table 4:** Characteristics of patients with BAD and LD in LSA and PPA territory

	BAD		*p*	LD		*p*
	LSA	PPA		LSA	PPA	
Number (%)	93 (65.5)	49 (34.5)^b^		94 (87.9)	13 (12.1)	
Male (%)	62 (66.7)	33 (67.3)	.935	65 (69.1)	7 (53.8)	.431
Age, years	59.81 ± 12.42	67.04 ± 9.37	.014^a^	64.16 ± 10.91	70.13 ± 11.09	.163
HbAlc, %	6.36 ± 1.63	7.34 ± 2.21	.054	6.26 ± 1.05	7.24 ± 1.72	.036^a^
TC, mmol/L	5.33 ± 1.50	5.26 ± 1.24	.634	5.27 ± 1.16	5.13 ± 1.13	.757
TRIG, mmol/L	2.01 ± 0.90	1.96 ± 0.94	.883	2.13 ± 1.53	1.99 ± 1.36	.821
HDL‐C, mmol/L	1.17 ± 0.26	1.18 ± 0.30	.915	1.17 ± 0.31	1.19 ± 0.20	.895
LDL‐C, mmol/L	3.84 ± 1.11	3.72 ± 0.98	.660	3.33 ± 0.93	3.29 ± 0.78	.910
LDL‐C/HDL‐C ratio	3.37 ± 1.01	3.25 ± 0.88	.632	2.98 ± 0.94	2.82 ± 0.73	.666
ApoAI, g/L	1.33 ± 0.30	1.33 ± 0.23	.932	1.29 ± 0.19	1.32 ± 0.26	.662
ApoB, g/L	0.96 ± 0.28	0.93 ± 0.29	.528	0.95 ± 0.29	0.95 ± 0.36	.996
ApoB/ApoAI ratio	0.75 ± 0.23	0.69 ± 0.17	.273	0.74 ± 0.22	0.72 ± 0.22	.741

BAD, branch atheromatous disease; LD, lipohyalinotic degeneration; LSA, lenticulostriate arteries; PPA, paramedian pontine arteries; HbAlc, Hemoglobin Alc; TC, total cholesterol; TRIG, triglyceride; HDL‐C, high‐density lipoprotein cholesterol; LDL‐C, low‐density lipoprotein cholesterol; ApoAI, apolipoprotein AI; ApoB, apolipoprotein B.

a *p* < .05.

b *p* < .001 compared with LD group in PPA territory.

## 4 Discussion

Previous research has shown that ischemic stroke is closely associated with metabolic risk factors, including hyperglycemia, hyperlipidemia, and hyperhomocysteinemia (Clarke et al., 1991). The association of homocysteine levels with PAD pathogenesis and progression has been demonstrated (Men et al., 2013). However, there is limited data on the impact of the lipids or glucose on PAD. In the present study, we focused on the potential risk factors, lipid indices and glycometabolism status and investigated the pathogenesis of first‐ever penetrating artery infarction.

Inflammation has a crucial role in the development of atherosclerosis, and the pathogenesis of stroke (Rost et al., 2001; Vila et al., 2000; Yilmaz et al., 2006). LDL‐C is an important pro‐inflammatory mediator in the oxidative process, After oxidization, LDL becomes more pro‐inflammatory. In the present study, we found that the level of LDL‐C and the LDL‐C/HDL‐C ratio were higher in the BAD group than in the LD group. The increase in LDL‐C/HDL‐C ratio could be mainly attributed to the elevated level of LDL‐C according to the data analysis. The elevated LDL‐C levels in patients with BAD may indicate a stronger oxidative reaction because of different pathogeneses, resulting in larger infarct volume. Our findings suggest that a high level of LDL‐C is a significant pathogenetic factor that can differentiate the clinical subtype of PAD. The exact cause of the increased proportion of progressive stroke in the BAD group remains unclear. We speculate that: In the BAD, the high level of LDL‐C could release large amounts of pro‐inflammatory cytokines, aggravate the atherosclerotic stenosis, contribute to the rupture of atherosclerotic plaques or expansion of thrombus located at the proximal end of the penetrating artery. Furthermore, although, currently, there is no consensus as to whether BAD should be included either among small vessel or among large vessel intracranial disease; our result may support that BAD pathogenesis and progression has a closer relationship with atherosclerosis and inflammation than LD, In fact, the multiple mechanisms LD caused by, such as lipohyalinosis, hypoperfusion, microatheroma, arteriosclerosis, and cardioembolic occlusion. By studying, we suggest the roles for LDL in the pathogenesis of LD occurrence account for the weak association between LDL and the risk of LD. Further research is necessary to elucidate the relationship between BAD and LD.

Our finding of the relationship between LDL‐C levels and progressive stroke has been supported by substantial clinical evidence. It has been reported that the concentration of plasma oxidized LDL‐C increases in the acute phase of all types of stroke (Nakase, Yamazaki, Ogura, Suzuki, & Nagata, 2008), indicating that oxidized LDL‐C participates in stroke development and progression. An animal experiment has confirmed that an increased concentration of LDL‐C in the arterial wall may be an early indication of lesion formation and a necessary step in the pathogenesis of the fatty streak lesion, leading to atherothrombosis (Schwenke & Carew, 1989). We speculate that in the process of ischemic stroke, the released pro‐inflammatory factors, including LDL‐C (more than 4.14 mmol/L), cause further lesion of the affected artery, which accelerates the expansion of thrombus, leading to the increase of infarct volume and progression and promote neurological deterioration. Accordingly, the LDL‐C level of patients with acute ischemic stroke should be strictly controlled with medications.

However, the association of lipids with ischemic stroke and its different subtypes remains controversial. Previous study reported that the triglyceride and non‐HDL‐C were associated with large artery atherosclerotic (LAA) stroke (Bang, Saver, Liebeskind, Pineda, & Ovbiagele, 2008). The elevated ApoB/ApoAI ratio was a predictor of ICAS in acute ischemic stroke (Park et al., 2011). However, in our study, no significant difference was found in the ApoB/ApoAI ratio between BAD and LD, and elevated LDL‐C levels may have the higher efficiencies for predicting BAD than any other lipid cholesterol parameters. Thus, studies are required to further investigate the relationship. Besides, several studies have reported that hyperglycemia is associated with progression in acute ischemic stroke (Nakase, Yoshioka, Sasaki, & Suzuki, 2013; Tanaka et al., 2013). But, our results revealed that the level of HbA1c was not statistically associated with progression in acute ischemic stroke, while there was a trend toward progression in BAD, The relatively small sample size may have limited the statistical power of the study.

Futhermore, Our findings also demonstrated that the LSA infarction was most common subtypes in PAI, the PPA infarction was more prevalent in the BAD. Our findings agree with previous studies (Bassetti, Bogousslavsky, Barth, & Regli, 1996; Kataoka, Hori, Shirakawa, & Hirose, 1997) which showed that BAD was etiologically the most common explanation for isolated pontine infarctions. The possible mechanism may be related with the specific vasculature and hemodynamics in pons. Besides, This may be reasonable to presume that the LSA infarction was caused by atheromatous changes at the origins or proximal portions of lenticulostriate arteries and lipohyalinotic degenerative changes of the distal small perforating arteries. However, The mechanism for PPA infarction was predominantly seems to be atheromatous changes at the origins or proximal portions of pontine penetrating arteries. Li et al. (2013) reported that the level of HbAlc may be associated with stroke severity and progression in brainstem infarctions. Our findings suggest that age at onset and HbA1c are the common risk factors for PPA infarction. The atherosclerotic change of small arteries should be differently altered by aging and glycometabolism status factors between the middle cerebral artery and the basilar artery. Previous studies, such as Kim et al. (2012) have reported that diabetic atherosclerosis is the most common vascular risk factors for posterior circulation ischemia, which was consistent with our results, However, the mechanisms involved remains uncertain. The relationship between age at the onset and PPA, LSA infarction has been studied, however, yielded conflicting results. Subramanian et al. (2009) reported age seems to be closely associated with anterior circulation stroke.

There are several limitations to our study. First, intracranial vessels were not assessed with adequately uniform vascular imaging, therefore, the study was unable to compare the frequency of underling parent artery disease in patients with progression versus those without progression, and in patients with suspected BAD versus those with LD infarctions; Secondly, compared with LDL‐C, oxidized LDL‐C is a better biomarker in reflecting the oxidative activity of patients; however, limited by the technical conditions, we were not able to detect the plasma oxidized LDL‐C. Thirdly, because a limited number of patients met our criteria for inclusion in the study, subgroups were not set up for validation of the results. Furthermore, bias is inevitable in retrospective studies.

In conclusion, Our findings supported the LDL‐C as the predictive marker of the pathogenesis and progression of intracranial BAD. The LSA infarction was the more frequently observed subtypes in PAI. PPA infarction was more often associated with BAD. Age at the onset and HbA1c were the major risk factors favoring PPA infarction of first‐ever PAI.

## Conflict of Interest

The authors declare that they have no conflict of interest.

## References

[brb3694-bib-0001] Bang, O. Y. , Saver, J. L. , Liebeskind, D. S. , Pineda, S. , & Ovbiagele, B. (2008). Association of serum lipid indices with large artery atherosclerotic stroke. Neurology, 70, 841–847. 18160673 10.1212/01.wnl.0000294323.48661.a9

[brb3694-bib-0002] Bassetti, C. , Bogousslavsky, J. , Barth, A. , & Regli, F. (1996). Isolated infarcts of the pons. Neurology, 46, 165–175. 8559368 10.1212/wnl.46.1.165

[brb3694-bib-0003] Baumgartner, R. W. , Sidler, C. , Mosso, M. , & Georgiadis, D. (2003). Ischemic lacunar stroke in patients with and without potential mechanism other than small‐artery disease. Stroke; a Journal of Cerebral Circulation, 34, 653–659. 10.1161/01.STR.0000058486.68044.3B 12624287

[brb3694-bib-0004] Caplan, L. R. (1989). Intracranial branch atheromatous disease: A neglected, understudied, and underused concept. Neurology, 39, 1246–1250. 2671793 10.1212/wnl.39.9.1246

[brb3694-bib-0005] Clarke, R. , Daly, L. , Robinson, K. , Naughten, E. , Cahalane, S. , Fowler, B. , & Graham, I. (1991). Hyperhomocysteinemia: An independent risk factor for vascular disease. The New England Journal of Medicine, 324, 1149–1155. 2011158 10.1056/NEJM199104253241701

[brb3694-bib-0006] Fisher, C. M. (1965). Lacunes: Small, deep cerebral infarcts. Neurology, 15, 774–784. 14315302 10.1212/wnl.15.8.774

[brb3694-bib-0007] Gao, S. , Wang, Y. J. , Xu, A. D. , Li, Y. S. , & Wang, D. Z. (2011). Chinese ischemic stroke subclassification. Frontiers in Neurology, 2, 6. 21427797 10.3389/fneur.2011.00006 PMC3052771

[brb3694-bib-0008] Kataoka, S. , Hori, A. , Shirakawa, T. , & Hirose, G. (1997). Paramedian pontine infarction. Neurological/topographical correlation. Stroke; a Journal of Cerebral Circulation, 28, 809–815. 10.1161/01.str.28.4.809 9099201

[brb3694-bib-0501] Kim, J. , Nah, H. W. , Park, S. M. , Kim, S. K. , Cho, K. H. , Lee, J. , … Lee, B. C. (2012). Risk factors and stroke mechanisms in atherosclerotic stroke: intracranial compared withextracranial and anterior compared with posterior circulation disease. Stroke, 43, 3313–3318. 23160885 10.1161/STROKEAHA.112.658500

[brb3694-bib-0009] Kwan, J. , & Hand, P. (2006). Early neurological deterioration in acute stroke: Clinical characteristics and impact on outcome. QJM: Monthly Journal of the Association of Physicians, 99, 625–633. 16905751 10.1093/qjmed/hcl082

[brb3694-bib-0010] Li, H. , Qiu, W. , Hu, B. , Kang, Z. , Wu, A. M. , Dai, Y. , … Lu, Z. (2013). Ischemic volumes and early neurologic deterioration in acute brainstem infarctions with Hemoglobin A. European Neurology, 70, 225–232. 24008404 10.1159/000351356

[brb3694-bib-0011] Lind, L. , Vessby, B. , & Sundstrom, J. (2006). The apolipoprotein B/AI ratio and the metabolic syndrome independently predict risk for myocardial infarction in middle‐aged men. Arteriosclerosis, Thrombosis, and Vascular Biology, 26, 406–410. 10.1161/01.ATV.0000197827.12431.d0 16306426

[brb3694-bib-0012] Matsuura, E. , Lopez, L. R. , Shoenfeld, Y. , & Ames, P. R. (2012). beta2‐glycoprotein I and oxidative inflammation in early atherogenesis: A progression from innate to adaptive immunity? Autoimmunity Reviews, 12, 241–249. 22569463 10.1016/j.autrev.2012.04.003

[brb3694-bib-0013] Men, X. , Li, J. , Zhang, B. , Zhang, L. , Li, H. , & Lu, Z. (2013). Homocysteine and C‐reactive protein associated with progression and prognosis of intracranial branch atheromatous disease. PLoS ONE, 8, e73030. 24039853 10.1371/journal.pone.0073030 PMC3770607

[brb3694-bib-0014] Nakase, T. , Yamazaki, T. , Ogura, N. , Suzuki, A. , & Nagata, K. (2008). The impact of inflammation on the pathogenesis and prognosis of ischemic stroke. Journal of the Neurological Sciences, 271, 104–109. 18479710 10.1016/j.jns.2008.03.020

[brb3694-bib-0015] Nakase, T. , Yoshioka, S. , Sasaki, M. , & Suzuki, A. (2013). Clinical evaluation of lacunar infarction and branch atheromatous disease. Journal of Stroke and Cerebrovascular Diseases: The Official Journal of National Stroke Association, 22, 406–412. 22133744 10.1016/j.jstrokecerebrovasdis.2011.10.005

[brb3694-bib-0016] Park, J. H. , Hong, K. S. , Lee, E. J. , Lee, J. , & Kim, D. E. (2011). High levels of apolipoprotein B/AI ratio are associated with intracranial atherosclerotic stenosis. Stroke; a Journal of Cerebral Circulation, 42, 3040–3046. 10.1161/STROKEAHA.111.620104 21868729

[brb3694-bib-0017] Rost, N. S. , Wolf, P. A. , Kase, C. S. , Kelly‐Hayes, M. , Silbershatz, H. , Massaro, J. M. , … Wilson, P. W. (2001). Plasma concentration of C‐reactive protein and risk of ischemic stroke and transient ischemic attack: The Framingham study. Stroke; a Journal of Cerebral Circulation, 32, 2575–2579. 10.1161/hs1101.098151 11692019

[brb3694-bib-0018] Schwenke, D. C. , & Carew, T. E. (1989). Initiation of atherosclerotic lesions in cholesterol‐fed rabbits. I. Focal increases in arterial LDL concentration precede development of fatty streak lesions. Arteriosclerosis, 9, 895–907. 2590067 10.1161/01.atv.9.6.895

[brb3694-bib-0502] Subramanian, G. , Silva, J. , Silver, F. L. , Fang, J. , Kapral, M. K. , Oczkowski, W. , … Investigators of the Registry of the Canadian Stroke Network. (2009). Risk factors for posterior compared to anterior ischemic stroke: an observational study of the Registry of the Canadian Stroke Network. Neuroepidemiology, 33, 12–16. 10.1159/000209282 19299902

[brb3694-bib-0019] Tabuchi, M. , Inoue, K. , Usui‐Kataoka, H. , Kobayashi, K. , Teramoto, M. , Takasugi, K. , … Matsuura, E. (2007). The association of C‐reactive protein with an oxidative metabolite of LDL and its implication in atherosclerosis. Journal of Lipid Research, 48, 768–781. 17261875 10.1194/jlr.M600414-JLR200

[brb3694-bib-0020] Tanaka, R. , Ueno, Y. , Miyamoto, N. , Yamashiro, K. , Tanaka, Y. , Shimura, H. , … Urabe, T. (2013). Impact of diabetes and prediabetes on the short‐term prognosis in patients with acute ischemic stroke. Journal of the Neurological Sciences, 332, 45–50. 23810779 10.1016/j.jns.2013.06.010

[brb3694-bib-0021] Vila, N. , Castillo, J. , Davalos, A. , & Chamorro, A. (2000). Proinflammatory cytokines and early neurological worsening in ischemic stroke. Stroke; a Journal of Cerebral Circulation, 31, 2325–2329. 10.1161/01.str.31.10.2325 11022058

[brb3694-bib-0022] Yamamoto, Y. , Ohara, T. , Hamanaka, M. , Hosomi, A. , Tamura, A. , & Akiguchi, I. (2011). Characteristics of intracranial branch atheromatous disease and its association with progressive motor deficits. Journal of the Neurological Sciences, 304, 78–82. 21402390 10.1016/j.jns.2011.02.006

[brb3694-bib-0023] Yamamoto, Y. , Ohara, T. , Nagakane, Y. , Tanaka, E. , Morii, F. , & Koizumi, T. (2010). Concept of branch atheromatous disease (BAD) and its clinical significance. Rinsho Shinkeigaku = Clinical Neurology, 50, 914–917. 21921508 10.5692/clinicalneurol.50.914

[brb3694-bib-0024] Yilmaz, G. , Arumugam, T. V. , Stokes, K. Y. , & Granger, D. N. (2006). Role of T lymphocytes and interferon‐gamma in ischemic stroke. Circulation, 113, 2105–2112. 16636173 10.1161/CIRCULATIONAHA.105.593046

